# Clinical utility of cardiac troponin measurement in COVID-19 infection

**DOI:** 10.1177/0004563220921888

**Published:** 2020-04-27

**Authors:** David C Gaze

**Affiliations:** ^1^Clinical Biochemistry, University of Westminster, London, UK; ^2^Clinical Blood Sciences, South West London Pathology, St George’s Healthcare NHS Foundation Trust, London, UK

**Keywords:** Troponin, analytes, Covid-19, SARS-Cov-2, cardiac troponin I, cardiac troponin T

## Abstract

The novel coronavirus SARS-CoV-2 causes the disease COVID-19, a severe acute respiratory syndrome. COVID-19 is now a global pandemic and public health emergency due to rapid human-to-human transmission. The impact is far-reaching, with enforced social distancing and isolation, detrimental effects on individual physical activity and mental wellbeing, education in the young and economic impact to business. Whilst most COVID-19 patients demonstrate mild-to-moderate symptoms, those with severe disease progression are at a higher risk of mortality. As more is learnt about this novel disease, it is becoming evident that comorbid cardiovascular disease is associated with a greater severity and increased mortality. Many patients positive for COVID-19 demonstrate increased concentrations of cardiac troponin, creating confusion in clinical interpretation. While myocardial infarction is associated with acute infectious respiratory disease, the majority of COVID-19 patients demonstrate stable cTn rather than the dynamically changing values indicative of an acute coronary syndrome. Although full understanding of the mechanism of cTn release in COVID-19 is currently lacking, this mini-review assesses the limited published literature with a view to offering insight to pathophysiological mechanisms and reported treatment regimens.

## Introduction

The seventh coronavirus known to infect humans is currently proliferating in humans at an alarming rate. The latest virus named SARS-CoV-2 (previously 2019-nCov) causes the disease COVID-19, a severe acute respiratory syndrome (SARS). Following the initial outbreak in Wuhan, China in late 2019, the World Health Organization (WHO) now considers SARS-CoV-2 a pandemic human viral infection.

At the time of writing (25 March 2020, 12:30), the live situation dashboard of the WHO (https://experience.arcgis.com/experience/685d0ace521648f8a5beeeee1b9125cd) reports 375,498 global cases and 16,362 deaths in 195 countries, areas or territories. There are 6,654 confirmed cases and 335 deaths in the United Kingdom.

There has been speculation that the virus was a result of genetic manipulation; however, SARS-CoV-2 more likely originated by natural selection in an animal source. The current SARS-CoV-2 virus demonstrates similar genetics to bat SARS-CoV like coronaviruses.^1^ There is, however, no evidence of direct bat-to-human transmission, suggesting an intermediate animal host is involved. This follows similar zoonotic infection routes of other coronaviruses entering into the human population.

**Figure 1.**
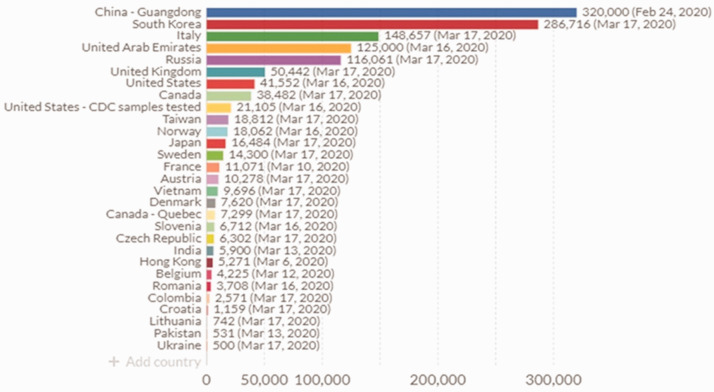
Global utilization of COVID-19 testing (source: https://ourworldindata.org/covid-testing).*Note:* Please refer to the online version of the article to view the figure in colour.

There has been a rapid response from the *in vitro* diagnostic industry to develop assays for SARS-CoV-2. These have migrated into UK laboratories at a much faster rate (50,442 tests on 18 March 2020) than in the US, due in part to stringent Food and Drug Administration regulations (Figure 1). Real-time reverse transcription polymerase chain reaction (RT-PCR) is used for SARS-CoV-2 RNA viral detection in upper and lower respiratory specimens, and serological analysis of anti-COVID-19 antibodies by automated immunoassays can be used for disease surveillance. The preferred testing is by molecular diagnosis of COVID-19 by real-time RT-PCR, such as the RdRp gene assay, which amplifies a conserved region of the RNA-dependent RNA polymerase gene that is specific to SARS-CoV-2, which has been used for confirmation of this disease by Public Health England laboratories. In addition, oligonucleotide primers and probes selected from regions of the virus nucleocapsid (N) gene are also included in the panel. In confirmed COVID-19 cases, the laboratory testing should be repeated to demonstrate viral clearance prior to healthcare discharge.

## Incubation, transmission and clinical presentation

The incubation period has been suggested to be approximately five days.^2^ Transmission is dependent on variable individual infectiousness, population density and spatial distance. The virus is transmitted primarily in respiratory aerosols and by indirect contact with contaminated surfaces. Faecal analysis detecting viral RNA also suggests a faecal–oral route of transmission.^3^

The clinical presentation and severity of symptoms is case-dependent. The clinical characteristics in the Chinese population have been recently documented from 1099 cases.^4^ The virus has infected more males than females, and severity is associated with older age. The common symptoms are fever and a persistent non-productive cough, although many present without fever and often with mild symptoms. The vast majority (>85%) do not demonstrate chest radiographic abnormalities, but ground-glass opacity and bilateral shadowing have been demonstrated on computer tomography in severe cases.

## Laboratory findings

Evidence from the Chinese cohort suggests prominent lymphocytopenia occurs in 83% of cases, with thrombocytopenia in 36% and leukopenia in 34%. Biochemically, patients demonstrate high concentrations of C-reactive protein (CRP) and less common elevations in liver enzymes (aspartate aminotransferase and alanine aminotransferase), creatine kinase (CK) and D-dimer.^4^ Furthermore, in a systematic analysis of 11 articles, Lippi and Plebani^5^ have documented laboratory abnormalities reported in cases of COVID-19. Patients may also present with decreased albumin, or increases in lactate dehydrogenase, total bilirubin, creatinine, procalcitonin and also cardiac troponin and natriuretic peptides.

## Cardiac troponin elevations in COVID-19

Previous influenza infection epidemics have been associated with myocardial infarction, myocarditis and exacerbated heart failure.^6^ These comorbid conditions contribute to significant mortality. Previous coronarvirus epidemics such as SARS in 2002 and Middle East Respiratory Syndrome (MERS) were associated with tachycardia, bradycardia, cardiomegaly, cardiac arrest, sub-clinical diastolic impairment and acute-onset heart failure.^7–11^

**Figure 2.**
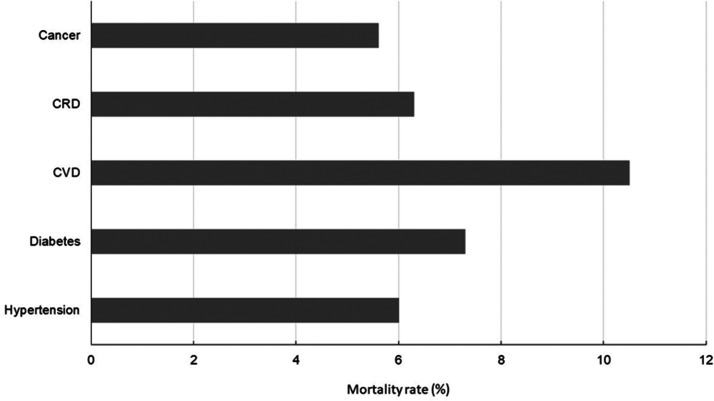
COVID-19 mortality rate in patients with pre-existing diseases (data source: Johns Hopkins University Centre for Systems Science and Engineering). CRD: chronic respiratory disease; CVD: cardiovascular disease.

COVID-19 is characterized by pneumonia and persons with underlying cardiovascular disease associated with hypertension, diabetes, coronary artery disease or cerebral vascular disease are at higher risk of developing the severest from of the disease and demonstrate the highest rate of mortality (Figure 2). Cardiac complications include the development of incident heart failure, acute coronary syndrome (ACS) and arrhythmia, all of which are associated with elevation in cTn^12^ especially when using high-sensitivity immunoassays and confer poor prognosis. Elevations in cTn are common in those with acute infectious respiratory disease and increases correlate with the severity of infection.^13^

Evidence of COVID-19-associated increases in circulating cardiac troponin T (cTnT) and cardiac troponin I (cTnI) above the 99th percentile reference limit are emerging in the literature.^14–17^ Detectable cTnI has been observed in most COVID-19 patients. In a retrospective cohort analysis, cTnI was significantly elevated in 54 subjects who died compared with 137 survivors (median [IQR] cTnI 22 [5.6–83.1] ng/L vs. 3 [1.1–5.5] ng/L, *P*≤0.0001).^18^

The mechanism of cTn elevation in COVID-19 infection is not fully understood. Elevations are likely to reflect non-coronary disease rather than acute coronary disease such as myocardial infarction.^19^ The underlying pathophysiology is suggestive of a cardio-inflammatory response, as many critically ill COVID-19 patients demonstrate concomitant elevations in acute phase reactants such as CRP and the natriuretic peptides. This may present clinically as fulminant myocarditis.

In one case report,^20^ a 37-year-old male presented with a three-day history of chest pain and dyspnoea. Electrocardiographic changes suggested an ST-segment elevation acute myocardial infarction, and cTnT was substantially elevated at >10,000 ng/L (99th percentile reference limit <14 ng/L), with concomitant elevations in CK and B-type natriuretic peptide. The initial working diagnosis was ACS. Subsequent CT coronary angiography revealed no evidence of coronary stenosis. A sputum sample was assayed for 13 viral nucleic acids, of which only coronavirus was positive. The working diagnosis changed to coronavirus fulminant myocarditis with cardiogenic shock and pulmonary infection. The patient was successfully treated with glucocorticoid and human Ig and cTnT decreased to 220 ng/L by one week and 21 ng/L by three weeks.

A further mechanism for consideration involves angiotensin converting enzyme 2 (ACE2), which is expressed in myocardial tissue. SARS-CoV-2 binds cells expressing ACE2.^21^ Binding of the virus can down-regulate ACE2 intracellular pathways and mediate inflammation and oedema, contributing to respiratory failure.^22^ In theory, this could have a potential impact on patients taking ACE inhibitors (ACEi), resulting in greater risk of acquiring COVID-19 infection and increased severity of disease. However, at present, the European Society of Cardiology has highlighted a lack of scientific evidence regarding COVID-19 infection in patients on ACEi or angiotensin receptor blockers and supports continuation of antihypertensive therapy in patients with confirmed infection (ESC.org).

## Concluding summary

The epidemiology of COVID-19 infection is evolving rapidly. As new cases are identified, understanding of clinical and diagnostic presentations is being refined. Cardiac biomarkers, in particular cTn and natriuretic peptides, are commonly elevated in patients with COVID-19 disease. As with many other non-ACS pathologies, elevation of cTn is associated with disease severity and poor prognosis. With the fast-moving development of disease progression across the globe and with better understanding of the mechanisms of cardiovascular complications in COVID-19, cardiac biomarkers can be utilized as a metric of a worsening clinical scenario or as an indicator of improving response due to cardioprotective intervention.
